# Biomarker Potential of *LINC00313* in Head and Neck Squamous Cell Carcinoma: Correlation with Epithelial-Mesenchymal Transition and Immune Cell Infiltration

**DOI:** 10.7150/ijms.93044

**Published:** 2024-03-31

**Authors:** Yikun Ju, Fang Zhu, Bairong Fang

**Affiliations:** 1 Department of Plastic and Aesthetic (Burn) Surgery, the Second Xiangya Hospital, Central South University, Changsha, Hunan, China.; 2 NHC Key Laboratory of Human Stem Cell and Reproductive Engineering, Institute of Reproductive and Stem Cell Engineering, School of Basic Medical Science, Central South University, Changsha, Hunan, China.

**Keywords:** HNSC, *LINC00313*, EMT, Immunotherapy, Prognosis.

## Abstract

Although *LINC00313* is dysregulated in several tumors, its role in head and neck squamous cell carcinoma (HNSC) is not fully understood. The aim of this study was to analyze the role of *LINC00313* in HNSC. The clinical information and *LINC00313* expression data of HNSC were mined from the TCGA/GEO/cbioportal database. The correlation between *LINC00313* expression and immune cell infiltration in HNSC tumors was analyzed by bioinformatics and gene enrichment analysis was performed. *LINC00313* was silenced in HNSC cell lines, and changes at the genetic and molecular levels were verified through qRT-PCR and Western blotting. The researchers also validated its functional phenotype through a series of cell function experiments. The results showed that overexpression and copy number variation of *LINC00313* in HNSC were associated with poorer prognosis. In addition, *LINC00313* expression was significantly negatively correlated with immune cell infiltration. Silencing of *LINC00313* in HNSC cells significantly reduced the rate of cell migration. *LINC00313* may affect the progression of HNSC by regulating epithelial-mesenchymal transition. In conclusion, *LINC00313* is a potential biomarker of HNSC prognosis and a potential target for immunotherapy.

## Introduction

Head and neck squamous cell carcinoma (HNSC) originated from the anatomical structures of the head and the neck, and includes nasopharyngeal carcinoma, oral cancer, tonsil cancer, laryngeal cancer, and oropharyngeal cancer, etc. More than 600,000 new cases of HNSC are diagnosed every year 1, and the five-year survival rate is around 50% 2. Due to its anatomical location, HNSC severely affects the patient's appearance, eating, speech, and breathing. Currently, HNSC is treated by surgical resection followed by local radiotherapy and systemic chemotherapy. However, these treatment modalities often seriously affect the quality of life of patients, and have not been effective in improving survival rates 3. Therefore, it is critical to identify novel biomarkers for the early diagnosis of HNSC to improve therapeutic outcomes.

The non-coding regions of the genome can also generate functional transcripts that regulate gene expression through various mechanisms. The long noncoding RNAs (lncRNAs) are more than 200 nucleotides in length, and cannot encode a protein due to the lack of a complete open reading frame 4, 5. However, lncRNAs can regulate gene expression through chromosome silencing, genomic imprinting, chromatin modification, transcriptional activation, transcriptional interference, and intranuclear transport, which affect biological processes including cancer development 6-9. Several lncRNAs have been identified that are involved in the development of HNSC 10. Feng *et al.* 11 identified 658 differentially expressed lncRNA transcripts in oral squamous cell carcinoma (OSCC) and validated their oncogenic role. Furthermore, simultaneous overexpression of LINC00511 and LINC00668 in tongue squamous cell carcinoma (TSCC) and OSCC cells increased their proliferation rates 11. LncRNAs such as Metastasis Associated Lung Adenocarcinoma Transcript 1 (*MALAT1*), *H19*, *NAG7* and *LINC00467* also promote the metastasis of HNSC cells by recruiting proteins or regulating the expression of cognate miRNAs 12. In addition, the lncRNA *HOTAIR* is significantly overexpressed in OSCC and is therefore a potential diagnostic biomarker 13. Thus, lncRNAs are involved in the occurrence and development of HNSC and are clinically relevant.

It is of great clinical importance to find a new biomarker for responding to the prognostic characteristics of HNSC, predicting the efficacy of HNSC patients' response to treatment or intervention, and then personalizing the treatment for them.* LINC00313* is associated with the poor prognosis of lung cancer and promotes the tumorigenesis of papillary thyroid carcinoma 14, 15. In addition, *LINC00313* is a prognostic marker of gastric cancer 16. However, the role of *LINC00313* in HNSC has not been elucidated. Herein, we analyzed the expression and prognostic value of *LINC00313* in HNSC using The Cancer Genome Atlas (TCGA) data, and further validated its oncogenic role through functional assays. This study will provide a theoretical basis for the development of *LINC00313* as a predictive and prognostic biomarker for HNSC and help scholars in related fields to recognize the potential role played by *LINC00313* in HNSC.

## Materials & Methods

### TCGA and GEO data mining

Expression data of *LINC00313* in the TCGA-HNSC dataset was downloaded from the UCSC Xena database (https://xena.ucsc.edu/) 17. The survival curves and prognosis of patients were plotted using an online analysis tool available in the database 17. The expression data of *LINC00313* in the GEO-GSE29330 dataset 18 was downloaded from the GEO database (https://www.ncbi.nlm.nih.gov/geo/) and analyzed using the GEO2R online tool according to default parameters 19-21.

### Risk Factor Analysis

RNA-sequencing data (level 3) of 504 tumors and corresponding clinical information were obtained from TCGA dataset 17. The data of 270 tumors of GEO-GSE65858 of downloaded from the GEO database 19-22. Data of 28 tumors and corresponding clinical information were obtained from The International Cancer Genome Consortium (ICGC) dataset (https://dcc.icgc.org/releases/current/Projects) 23. RNA-sequencing expression (level 3) profiles and corresponding clinical information for HNSC were downloaded from the ICGC dataset (https://dcc.icgc.org/releases/current/Projects). Log-rank test was used to compare differences in survival between these groups. For Kaplan-Meier curves, p-values and hazard ratio (HR) with 95% confidence interval (CI) were generated by log-rank tests and univariate cox proportional hazards regression. The predictive accuracy and risk score of *LINC00313* were generated using R software (v.4.2.2) package “survival” (v3.4.0) through Hiplot Pro (https://hiplot.com.cn/), a comprehensive web service for biomedical data analysis and visualization 24. For Kaplan-Meier curves, p-values and hazard ratio (HR) with 95% confidence interval (CI) were generated by log-rank tests and univariate Cox proportional hazards regression Univariate and multivariate Cox regression analysis were performed to identify the proper terms to build the nomogram. The forest was used to show the P value, HR and 95% CI of each variable through 'forestplot' R package (version: 3.1.3, https://cran.r-project.org/web/packages/forestplot/index.html). A nomogram was developed based on the results of multivariate Cox proportional hazards analysis to predict the 5-year overall recurrence. The nomogram provided a graphical representation of the factors which can be used to calculate the risk of recurrence for an individual patient by the points associated with each risk factor through 'rms' R package (version:6.7-0, https://cran.r-project.org/web/packages/rms/index.html).

### Cell culture, siRNA transfection and qRT-PCR

The SCC-9 and Cal-27 cell lines were obtained from the Department of Stomatology, Second Xiangya Hospital, Central South University, and cultured as previously described 25. Both cell lines were previously purchased from the American Type Culture Collection cell bank, and primary cells obtained from humans were not involved in this study. All cells were cultured in a cell culture incubator at 37 ℃, 5% CO2, 95% humidity with Dulbecco's Modified Eagle Medium (DMEM) + 10% fetal bovine serum (FBS) + 1% penicillin-streptomycin. Transfection was performed at 60% cell density. The siRNAs were separately transfected into cells using Lipofectamine 3000. The siRNA targeting *LINC00313* was as follow: *LINC00313*-siRNA: GCTTCCTGGATTGCATAAA. The following primers were used for qRT-PCR: *LINC00313*, 5′-CCTGGATTGCATAAAGGCTC-3′ and 5′-GGATCTGCAGAAACACTCTC-3′; ACTB, 5′-TCACCAACTGGGACGACATG-3′ and 5′-GTCACCGGAGTCCATCACGAT-3′.

### Cell transwell migration experiments

We performed functional experiments using the cells after transfection with siRNA for 48 hours. Cells were digested using 0.25% trypsin for 2 mins, after which digestion was terminated using complete medium. Cells were washed twice using phosphate buffered saline (PBS). The cells were counted using a cell counter. The migration capacities of cells were determined by transwell assay. The cells at a density of 2 × 10^4^ cells/well were inoculated above the 8 µm chamber (NY-Corning, USA) with a medium containing 200 µL with a medium containing 2% FBS and below the chamber with a medium containing 15% FBS of 800 µL for 24 h. The wells were stained using 0.1% crystalline violet solution, after which photographs were taken under the microscope and five fields of view were selected for counting and analysis.

### Cell scratch migration experiment

After 48 h of transfection with siRNA, the cell density was over 90%. The cells were washed twice with PBS, and a straight line was drawn using a 10 μL tip. After washing twice again with PBS, pictures were taken under a microscope. After replacing the cell culture medium and incubating for 24h, pictures were taken again, and the cell migration rate was observed and calculated. The migration ability of the cells was assessed using ImageJ software 26.

### Western blotting

Cell precipitates were harvested after cell transfection for 72 hours and used to extract proteins for Western Blot. After which protein quantification was performed using a BCA (Bicinchoninic acid) protein quantification kit. Next, 30 µg cellular protein was added to each well of a 4-20% FuturePAGE gel (ACE Biotechnology, Nanjing, China). After electrophoresis, the proteins were transferred onto polyvinylidene fluoride (PVDF) membranes, blocked using 5% skim milk at room temperature for one hour. Followed by overnight incubation with primary antibody (1:1000, α-tubulin, E-cadherin, β-catenin, Slug and vinculin, Cell Signaling Technology, USA) at 4 degrees. The next day, after washing 3 times with TBST (Tris buffered saline + Tween), the secondary antibody (1:1000, HRP-linked anti-rabbit IgG, Cell Signaling Technology, USA) was incubated at 37 degrees for 1 hour. After washing 3 times again with TBST, images were taken and analyzed using the Vilber FUSION fx6.uedge imaging system.

### Signaling pathways correlation analysis

The signaling pathways related to *LINC00313* in HNSC were screened using the GSCA online tool (http://bioinfo.life.hust.edu.cn/GSCA/#/) based on the TCGA PanCancer Atlas HNSC queue data according to default parameters 27. The correlation between *LINC00313* and Epithelial-Mesenchymal Transition (EMT) was analyzed by the GEPIA2 online tool (http://gepia2.cancer-pku.cn/#index) based on the TCGA PanCancer Atlas HNSC queue data according to default parameters 28. Raw counts of RNA-sequencing data (level 3) and corresponding clinical information from HNSC were obtained from the TCGA and ICGC dataset. ClusterProfiler package (version: 3.18.0) in R was employed to enrich the Kyoto Encyclopedia of Genes and Genomes (KEGG) pathway 29. The KEGG pathway database is a comprehensive biochemical pathway database often used for studying various pathways (www.kegg.jp/kegg/kegg1.html) 30-32.

### Immunophenotypic correlation analysis

The RNA-sequencing expression (level 3) profiles, genetic mutation and corresponding clinical information for HNSC were downloaded from the TCGA dataset and the ICGC dataset 23. The R software ggstatsplot package (version 0.12.0, https://indrajeetpatil.github.io/ggstatsplot/) was used to draw the correlations between gene expression and immune score 33. The Tumor Immune Dysfunction and Exclusion (TIDE) algorithm was used to predict potential immunotherapy responses 34, 35. The correlations between *LINC00313* and immune-related molecules were analyzed by the GEPIA2 online tool (http://gepia2.cancer-pku.cn/#index) based on the TCGA PanCancer Atlas HNSC queue data according to default parameters 28. The drug sensitivity analysis based on multiple sets of HNSC data including the TCGA were used the BEST online tool (https://rookieutopia.com/app_direct/BEST/), within the scope of its authorization.

### Genomic variation analysis of *LINC00313*

The genomic variants of *LINC00313* and their correlation with clinicopathological features of HNSC were analyzed using the cBioportal online tool (https://www.cbioportal.org/) according to default parameters 36, 37. The data sets were the TCGA PanCancer Atlas HNSC cohort data and the TCGA Firehose Legacy HNSC cohort data.

### Statistical analysis

Statistical analysis was conducted using Graphpad or other related online tools. The difference between the two groups was compared using the student t-test. The survival curves were compared using Log-rank test. Correlation analysis was conducted using the Pearson method. P values less than 0.05 were considered statistically significant.

## Results

### *LINC00313* is aberrantly highly expressed in HNSC

**Figure 1:**
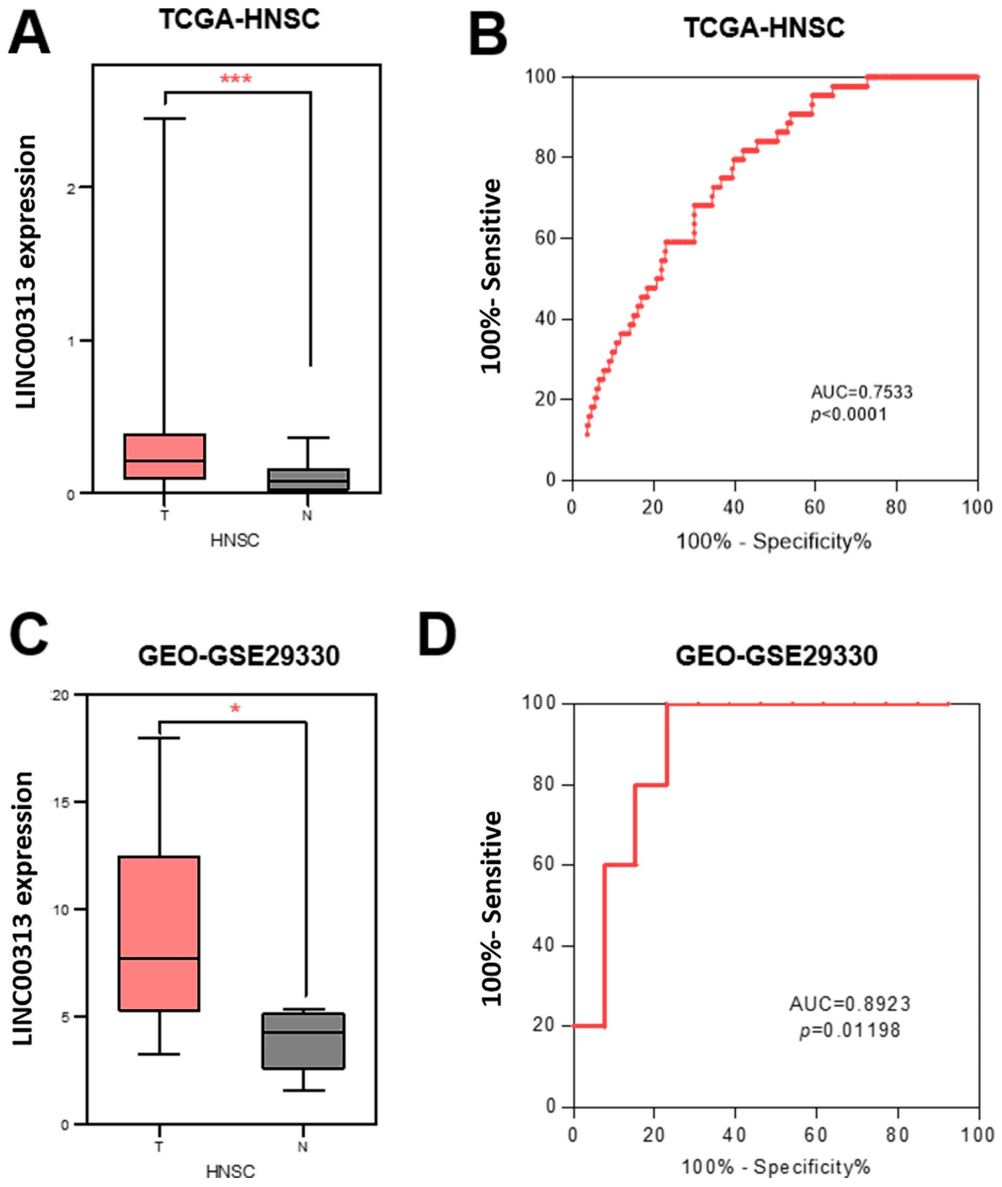
**The expression of *LINC00313* was higher in HNSC compared to normal tissues. (A)** Differential expression of *LINC00313* in HNSC and normal tissues based on the TCGA-HNSC dataset. **(B)** *LINC00313* expression was confirmed to be sensitive to HNSC by ROC curves based on the TCGA-HNSC dataset. **(C)** Differential expression of *LINC00313* in HNSC and normal tissues based on the GEO GSE29330 dataset. **(D)** *LINC00313* expression was confirmed to be sensitive to HNSC by ROC curves based on the GEO GSE29330 dataset. *: P-value < 0.05, ***: P-value < 0.001.

To determine the clinical value of *LINC00313* in HNSC, we downloaded its expression data and the related clinical data from TCGA-HNSC, GSE29330, and GSE42743 datasets. *LINC00313* was significantly upregulated in the 502 HNSC tumors relative to the 44 adjacent normal tissues from the TCGA-HNSC dataset **(Figure 1A)**. We used receiver operating characteristic (ROC) curve to predict its prediction accuracy and found that it has good prediction accuracy (AUC=0.7533, P<0.0001) **(Figure 1B)**. The area under curve (AUC) of *LINC00313* was greater than 0.5, indicating good predictive power, which is indicative of its diagnostic value. *LINC00313* was also significantly upregulated in the 13 HNSC tumors relative to the 5 adjacent normal tissues from the GSE29330 dataset **(Figure 1C)**. The ROC curve showed a good prediction accuracy also (AUC=0.8923, P=0.01198)** (Figure 1D)**. Similarly, we verified in the GSE65858 dataset that *LINC00313* expression was also higher in 24 pairs of tumors than in normal tissues** (Supplementary Figure 1A)**. Afterward, we analyzed the expression of *LINC00313* in HNSC patients with different clinical characteristics. The expression of *LINC00313* in HNSC is independent of gender** (Supplementary Figure 1B)**. We also found some interesting trends, although there were no statistically significant differences. Overall, *LINC00313* appears to be more highly expressed in the late tumor stages **(Supplementary Figure 1C)**. However, in the TNM stage of the tumor, we found that *LINC00313* appeared to be more highly expressed in the high-grade T stages, but the opposite trend was observed in the N stages and M stages **(Supplementary Figure 1D-F)**. Therefore, we hypothesize that LINC0313 may affect tumor progression primarily by promoting tumor cell migration, rather than invasion.

### *LINC00313* is a potential prognostic marker for HNSC

**Figure 2:**
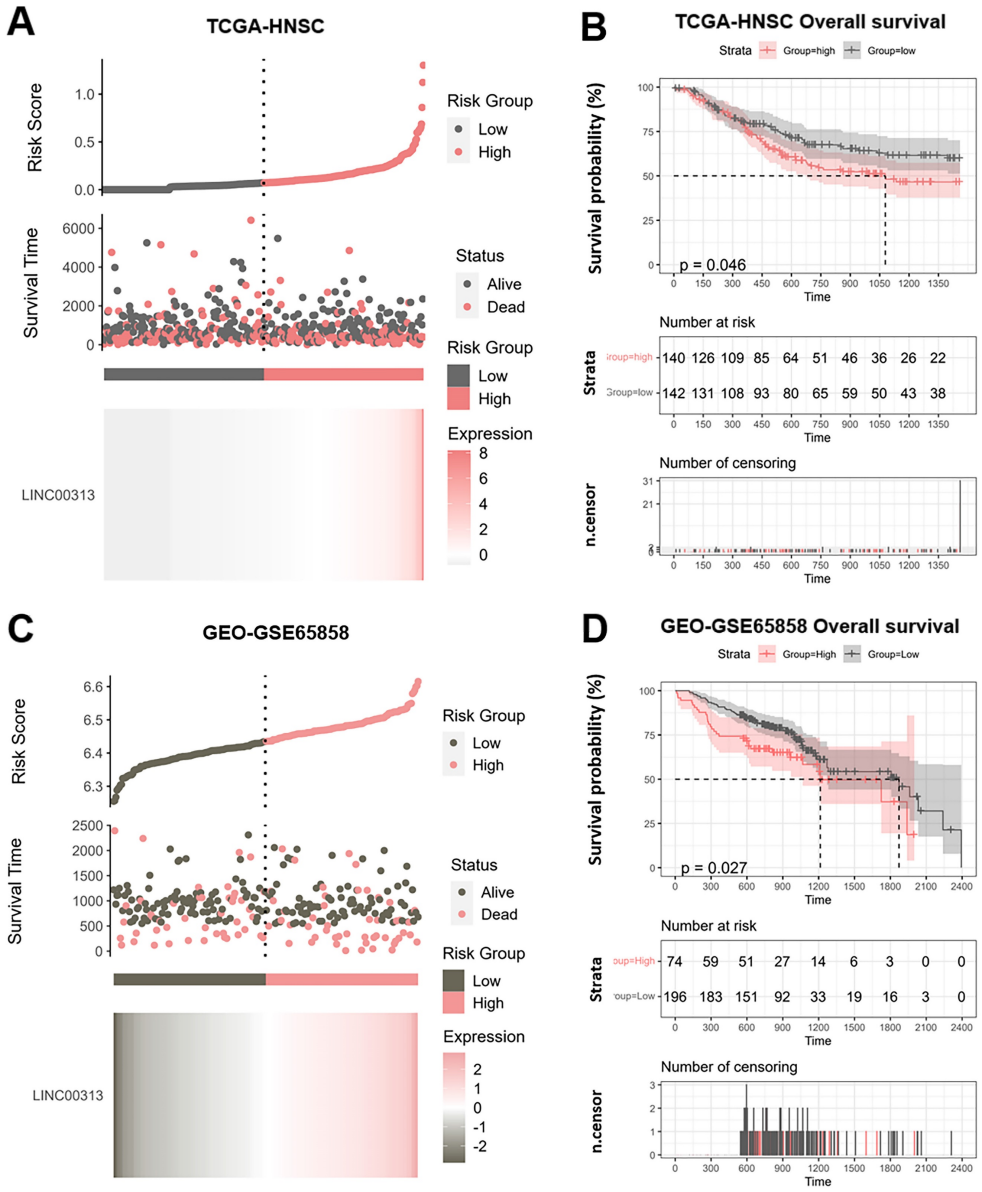
** Potential prognostic value of *LINC00313* in HNSC. (A&C)** Risk score curve showing survival of patients and expression profiles of *LINC00313* in low- and high-risk groups based on the TCGA-HNSC and GSE65858 dataset. The Riskscore, survival time and survival status of selected dataset. The top scatterplot represents the Riskscore from low to high. Different colors represent different groups. The scatter plot distribution represents the Riskscore of different samples correspond to the survival time and survival status. The buttom heatmap is the *LINC00313* expression from different HNSC samples. **(B&D)** *LINC00313* expression correlated with the overall survival rates based on the TCGA HNSC dataset and GSE65858 dataset.

We divided the patients into high-risk and low-risk groups according to the median risk score of *LINC00313* (those with expression above the median value were in the high-risk group and vice versa). We analyzed the relationship between *LINC00313* expression and survival time and survival status. We found that abnormally high expression of *LINC00313* was associated with a poorer prognosis based on TCGA-HNSC dataset **(Figure 2A)**. We plotted the Kaplan-Meier (KM) survival curves of association between *LINC00313* and overall survival (OS) of HNSC, where the different groups were tested by log-rank tests. The results showed lower OS survival in the high-risk group compared to the low-risk group (high-risk group 140 cases, low-risk group 142 cases) (P=0.046)** (Figure 2B)**. In addition, analysis of the HNSC data in the ICGC dataset also showed that patients with high expression of *LINC00313* had a lower overall survival rate (high-risk group 74 cases, low-risk group 196 cases) (P=0.026)** (Figure 2C, 2D).** We performed the same analysis in the ICGC database, showing the same trend. Still, probably due to the small sample size, it did not show a statistical difference (high-risk group 19 cases, low-risk group 9 cases) (P=0.077) **(Supplementary Figure 2A, 2B)**.

**Figure 3:**
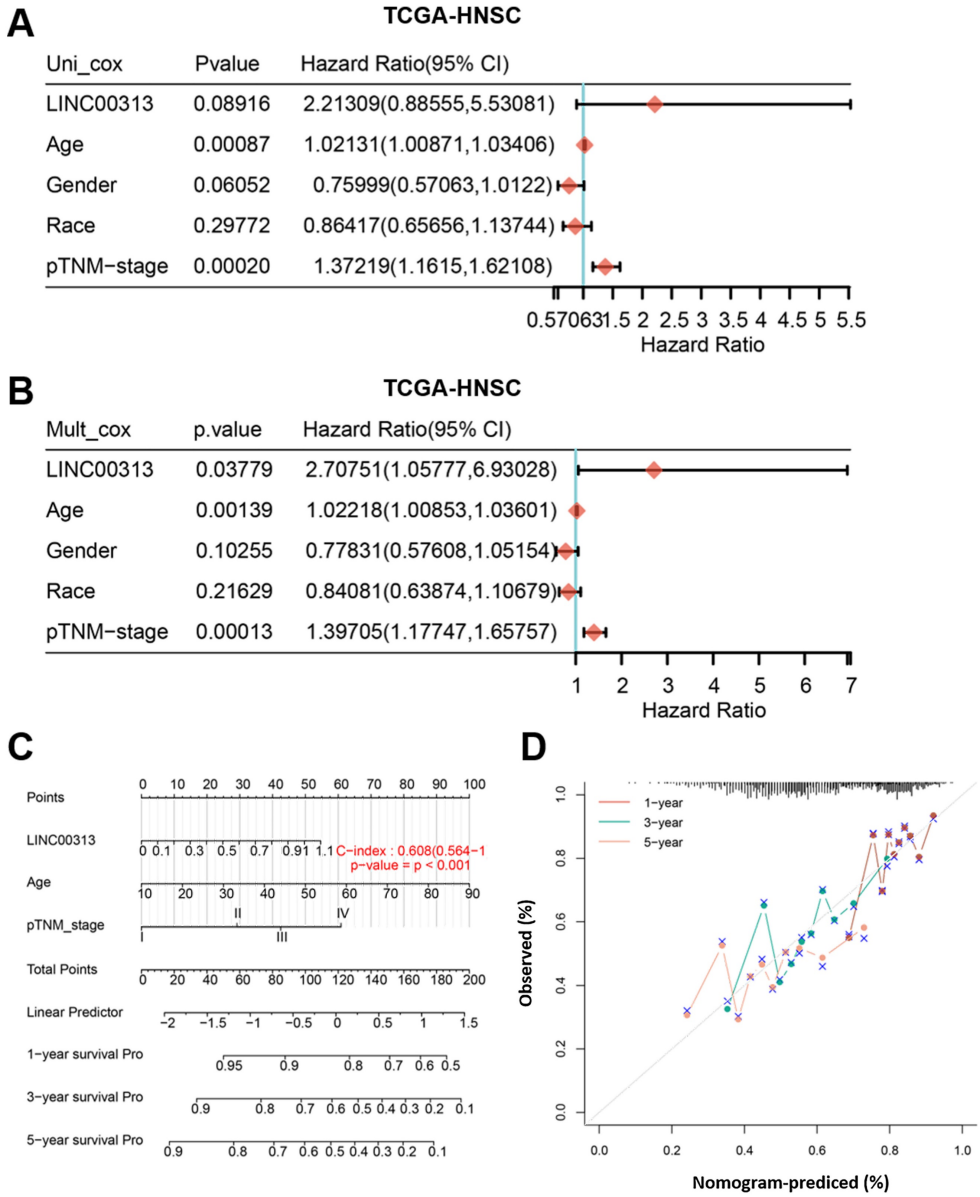
** The identification of independent prognostic factors for OS and the development of the nomogram in TCGA-HNSC. (A)** Forest plot presenting the univariate Cox regression analysis. **(B)** Forest plot presenting the multivariate Cox regression analysis. **(C)** Nomogram. **(D)** The calibration curves for predicting the correlation between 1-, 3- and 5-year overall survival and *LINC00313* expression in HNSC patients.

From multivariate Cox proportional hazard regression, *LINC00313* (P=0.03779, HR=2.70751, 95% CI=1.05777, 6.93028) and age (P=0.00139, HR=1.02218, 95% CI=1.00853, 1.03601) were identified as significant positive prognostic factors for pTNM-stage (P = 0.00013, HR = 1.39705, 95% CI = 1.17747-1.65757) **(Figure 3A)**. The univariate Cox proportional hazard regression showed in **Figure 3B**. A combination of marker *LINC00313* and the clinical feature age was selected as a prognostic model. Afterward, we developed a novel prognostic nomogram for HNSC patients based on the new model (*LINC00313* expression and age) with the C index of 0.608 (P <0.001, 95% CI = 0.564-1) which means 60.8% Calibration of prediction the 1, 3, and 5-years OS in the HNSC patients **(Figure 3C)**. Calibration curves of the nomogram are shown in **Figure 3D**. In summary, *LINC00313* is a valuable potential prognostic marker for HNSC.

### *LINC00313* promotes HNSC cell migration

**Figure 4:**
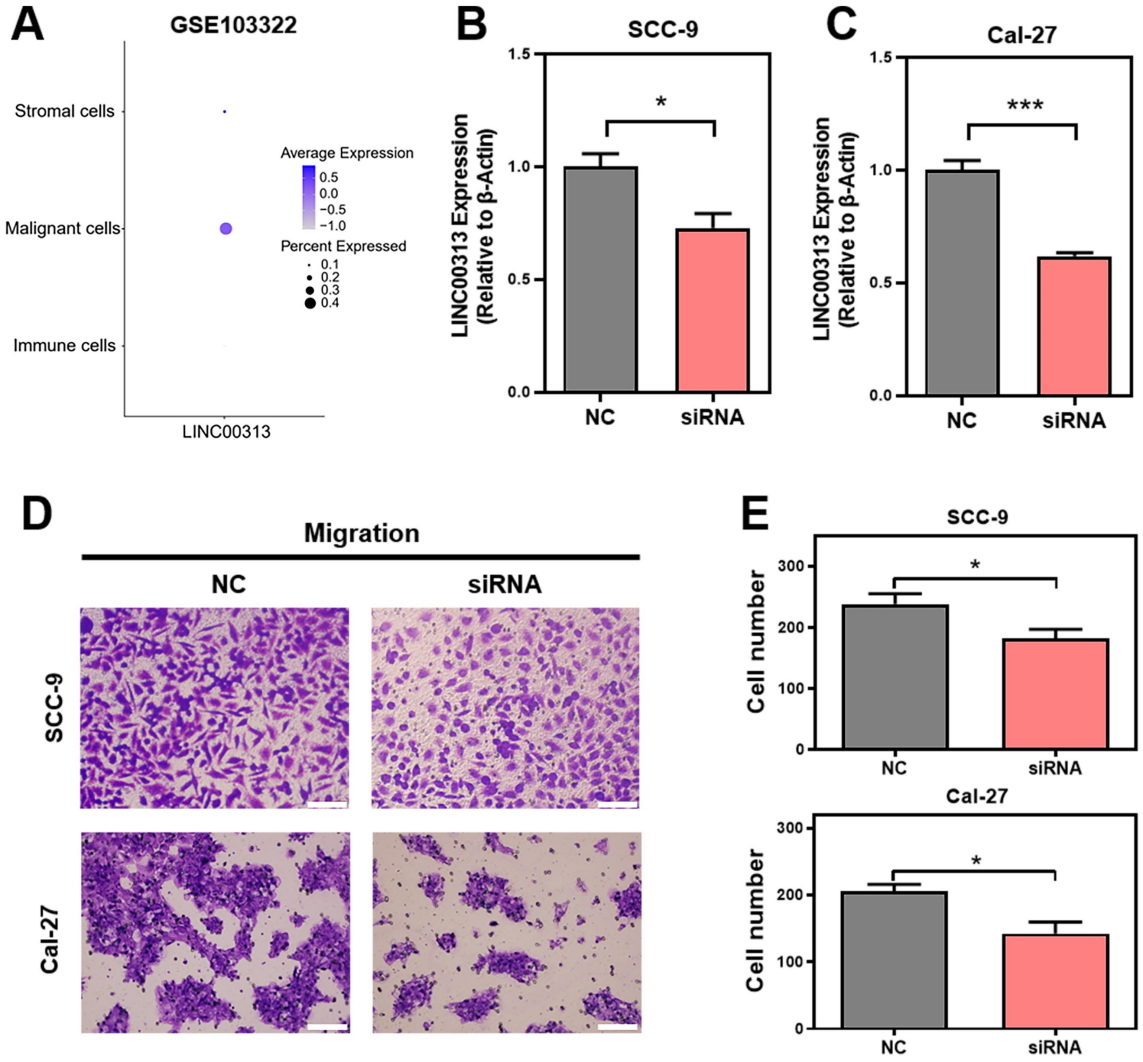
***LINC00313* promotes HNSC cell migration. (A)** The expression of *LINC00313* in various cell populations in HNSC. **(B&C)** The silencing efficiency of *LINC00313* in SCC-9 cell line and Cal-27 cell line. **(D)** Negative control and *LINC00313*-silenced cells transwells migration results in SCC-9 cell line and Cal-27 cell line. **(E)** Statistical analysis of the number of migrated cells. *: P-value < 0.05, **: P-value < 0.01, ***: P-value < 0.001.

Given the heterogeneity of tumor tissues, single-cell sequencing can facilitate gene expression analysis of specific cells. Therefore, we analyzed the expression of *LINC00313* in the different cell populations of HNSC tumors using the GSE103322 single-cell sequencing dataset. The expression levels of *LINC00313*, as well as the percentage of positively expressing cells, were significantly higher among the malignant cells **(Figure 4A)**. We designed an siRNA against *LINC00313* and transfected it into the cell lines of HNSC (reference method section). The silencing efficiency of *LINC00313* in SCC-9 **(Figure 4B)** and Cal-27 cell line **(Figure 4C)** were examined using qRT-PCR. We then examined the migratory ability of the two cell lines using transwell experiment and found that cells silenced with the siRNA signficantly reduced migration capacity compared to the control group **(Figure 4D, 4E)**. The results were statistically different. Meanwhile, we also performed cell scratch assays to aid in demonstrating the effect of *LINC00313* on cell migration ability. The results showed that the scratch migration ability of silenced *LINC00313* cells was significantly decreased compared with the control group, which was consistent with the results of the transwell migration assay **(Supplementary Figure 3)**. Consistent with our previous speculation, *LINC00313* may affect tumor progression primarily by enhancing tumor cell migration.

### Signaling pathways that *LINC00313* might regulate

**Figure 5:**
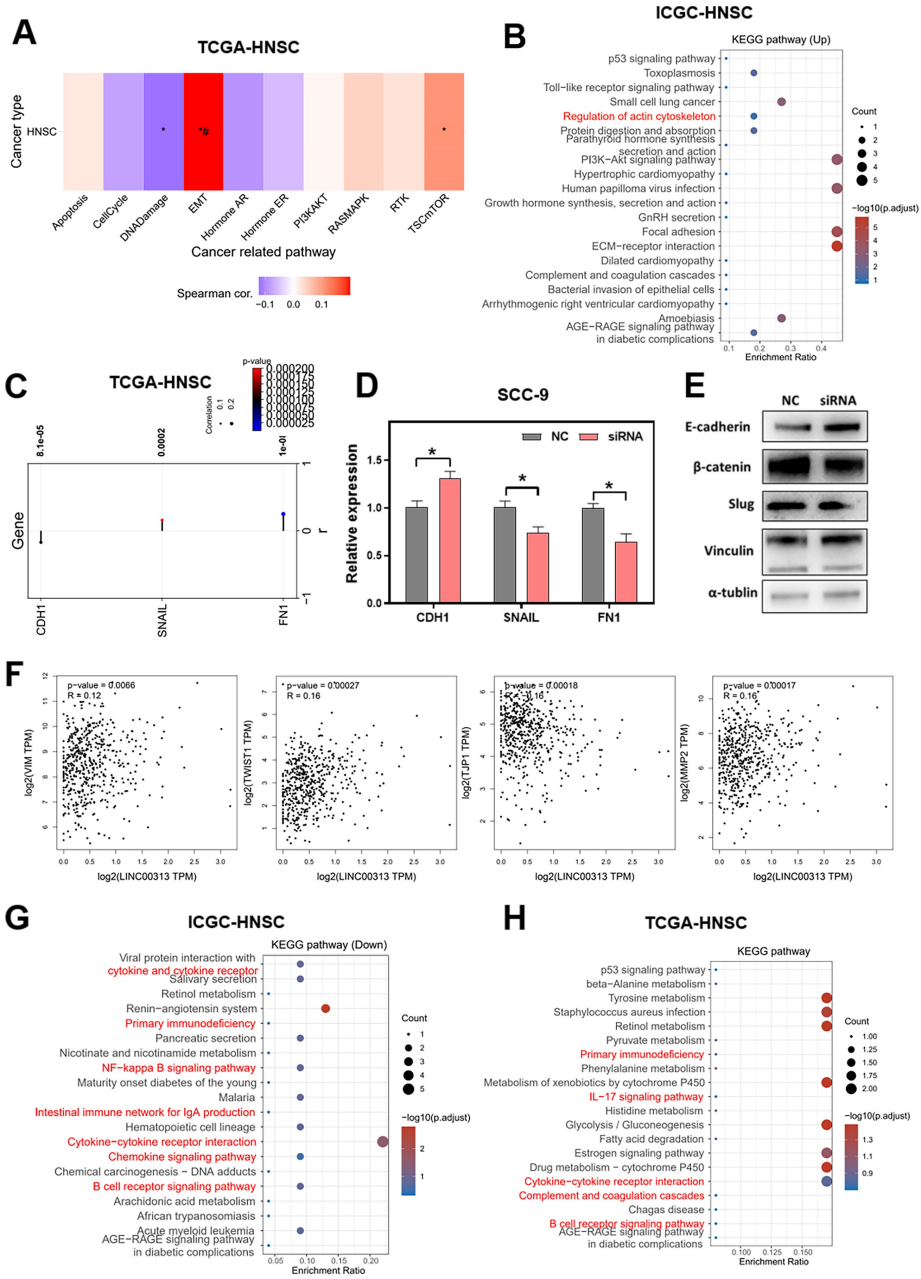
** Signaling pathways that *LINC00313* might regulate. (A)** The correlation between *LINC00313* and the activity of each signaling pathway based on the TCGA-HNSC dataset.** (B)** Signaling pathways differentially upregulated in the *LINC00313* high- and low-expression groups based on KEGG analysis. **(C)** Correlation of *LINC00313* expression with the expression of CDH1, SNAIL, and FN1. **(D)** The expressions of CDH1, SNAIL and FN1 on the control and *LINC00313*-silenced group in SCC9 cells were assessed using qRT-PCR. **(E)** Western blot results on *LINC00313*-silenced group and control group in SCC9 cells. **(F)** Correlation analysis of *LINC00313* expression with VIM, TWIST1, TJP1, and MMP2. **(G)** Signaling pathways differentially down-regulated in the *LINC00313* high- and low- expression groups based on KEGG analysis. **(H)** Signaling pathways differentially regulated in the tumors and normal tissues based on KEGG analysis. *: P-value < 0.05.

To explore the downstream signaling pathways regulated by *LINC00313*, we analyzed the correlation between *LINC00313* and the different pathways based on the ICGC-HNSC dataset and the TCGA-HNSC dataset. We compared the correlation of *LINC00313* with tumor-associated signaling pathways and found a significant correlation with Epithelial-Mesenchymal Transition (EMT)** (Figure 5A)**. Subsequently, we compared the differential KEGG signaling pathways between the high- and low-* LINC00313* expression groups based on the ICGC-HNSC dataset **(Figure 5B)**. The cytoskeleton regulation-related signaling pathways were significantly unregulated, such as “Regulation of actin cytoskeleton”** (Figure 5B)**.

Our further analysis showed a negative correlation between LNC00313 and the epithelial marker CDH1. In contrast, the expression level of *LINC00313* showed a significant positive correlation with the EMT assembly-related transcription factors *SNAIL* and the cytoskeletal assembly related molecule *FN1*** (Figure 5C).** Then, we have verified it using qRT-PCR. We found that the expression of CDH1 was higher in *LINC00313*-silenced SCC9 cells than control group, and the expression of *SNAIL* and *FN1* were lower. The experimental results are consistent with the previous analysis **(Figure 5D)**.

Subsequently, we validated the EMT-related proteins at the protein level using western blotting** (Figure 5E, original blots/gels are presented in Supplementary Figure 4)**. The results showed that, consistent with the previous bioinformatic prediction results and qRT-PCR, E-cadherin (CDH1) expression was upregulated in the *LINC00313*-silenced group. Also, we found that he expression of β-catenin and Slug were suppressed in the *LINC00313*-silenced group. Previous studies have shown that the Wnt/β-catenin signaling pathway affects tumor progression by influencing the expression of E*-cadherin*. Slug is an important transcription factor of the EMT pathway and is involved in EMT mainly through the regulation of E-cadherin. In addition, supplemental bioinformatic analyses based on the TGCA-HNSC data showed the correlation of other EMT molecules with *LINC00313*: VIM (P=0.0066, R=0.12), TWIST1 (P=0.00027, R=0.16), TJP1 (P=0.00018, R=-0.16), MMP2 (P=0.00017, R=0.16) **(Figure 5F)**.

Further, we compared the differentially down-regulated KEGG signaling pathways between the high- and low-* LINC00313* expression groups based on the ICGC-HNSC dataset **(Figure 5G)**. We found that many immune-related signaling pathways were enriched, such as “Primary Immunodeficiency”, “NF-kappa B signaling pathway”, “Cytokine-cytokine receptor interaction”, and “B cell receptor signaling pathway”. And compared the relevant differentially expressed KEGG signaling pathways in normal and tumor tissues based on the TCGA-HNSC dataset **(Figure 5H)**. The differentially genes between normal and tumor tissues from the TCGA-HNSC dataset were also mainly involved in “Primary Immunodeficiency”, “IL-17 signaling pathway”, “Cytokine-cytokine receptor interaction”, and “B cell receptor signaling pathway”. We found that the pathways aberrantly expressed in tumors overlapped with a significant portion of the *LINC00313* high-expression group. This suggests that the pathways affected by *LINC00313* are some important pathways associated with HNSC tumors.

### *LINC00313* expression level negatively correlates with immune infiltration in HNSC patients

**Figure 6:**
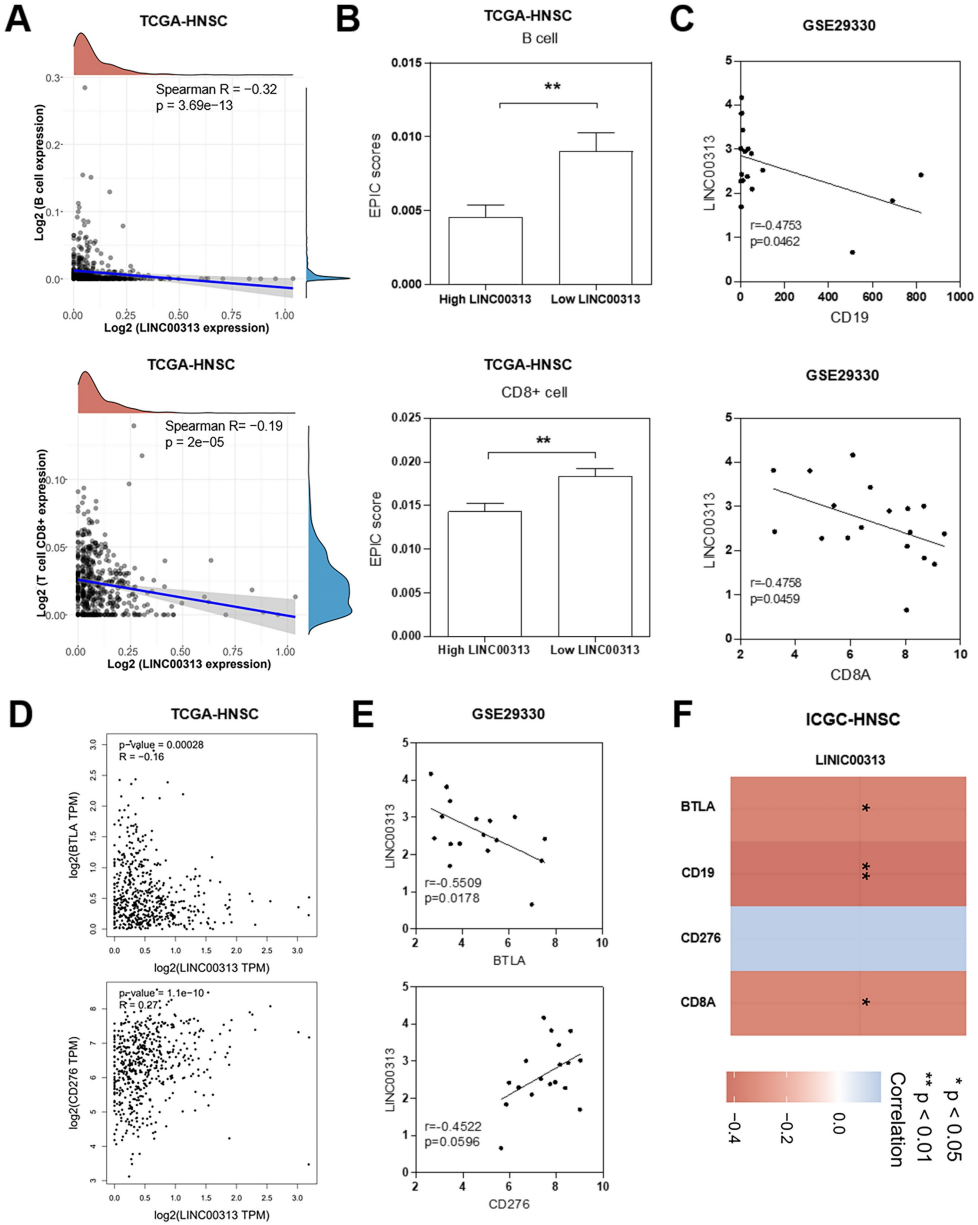
***LINC00313* expression level negatively correlates with immune infiltration in HNSC patients. (A)** Correlation between *LINC00313* expression and B cell and CD8+ T cell.** (B)** The EPIC scores of B cell and CD8+ T cell in *LINC00313* high- and low-expression groups. **(C)** Correlation between *LINC00313* and CD19, CD8A based on the GSE29330 dataset. **(D)** Correlation between *LINC00313* and BTLA, CD276 based on the TCGA-HNSC dataset, and **(E)** GSE29330 dataset. **(F)** Heatmap of immune checkpoint molecules associated with *LINC00313* expression. *: P-value < 0.05, **: P-value < 0.01.

We considered that *LINC00313* may affect the immune response of patients to HNSC, so we analyzed the correlation between *LINC00313* and immune infiltration. In tumor tissues, due to the infiltration of various cells in the tumor microenvironment, a variety of cell subpopulations such as tumor cells, infiltrating immune cells, etc. Firstly, we analyzed the correlation between *LINC00313* expression and immune cell expression. The results showed that *LINC00313* expression was inversely associated with the expression of B cells (Spearman R=-0.32, p=3.69e-13), and CD8+ T cells (Spearman R=-0.19, p=2e-05) **(Figure 6A)**. Afterward, we evaluated the EPIC scores of B cells and CD8+ T cells in different *LINC00313* expression groups. The results showed that the EPIC scores fraction of both B and CD8+ T cells was significantly lower in the *LINC00313* low-expression group than in the high-expression group (P<0.01)** (Figure 6B)**. CD19 is mainly distributed on the surface of B cells. CD8A is mainly distributed on the surface of CD8+ T cells. We validated our analysis again with the GEO-GSE29330 dataset and showed that *LINC00313* expression was also significantly negatively correlated with CD19 (r=0.4753, P=0.0462) and CD8A (r=0.4758, P=0.0459)** (Figure 6C).** BTLA is a CD28/B7 family member, expressed by most lymphocytes, which shares structural and functional similarity with CTLA-4 and PD-1. While, CD276 expression enables squamous cell carcinoma stem cells to evade immune surveillance and as an immunotherapy target for HNSC. We have analyzed the correlation between related molecules and *LINC00313* based on the three different datasets: TCGA-HNSC, GSE29330, and ICGC-HNSC. Further analysis of immune checkpoint molecules revealed that *LINC00313* was significantly negatively correlated with BTLA, CD8A, and CD19 and positively correlated with CD276** (Figure 6D, E, F).** These suggest that *LINC00313* may play an inhibitory effect on the infiltration and activation of immune cells, such as B cells and CD8+ T cells.

### *LINC00313* expression may be associated with HNSC immunotherapy response

**Figure 7:**
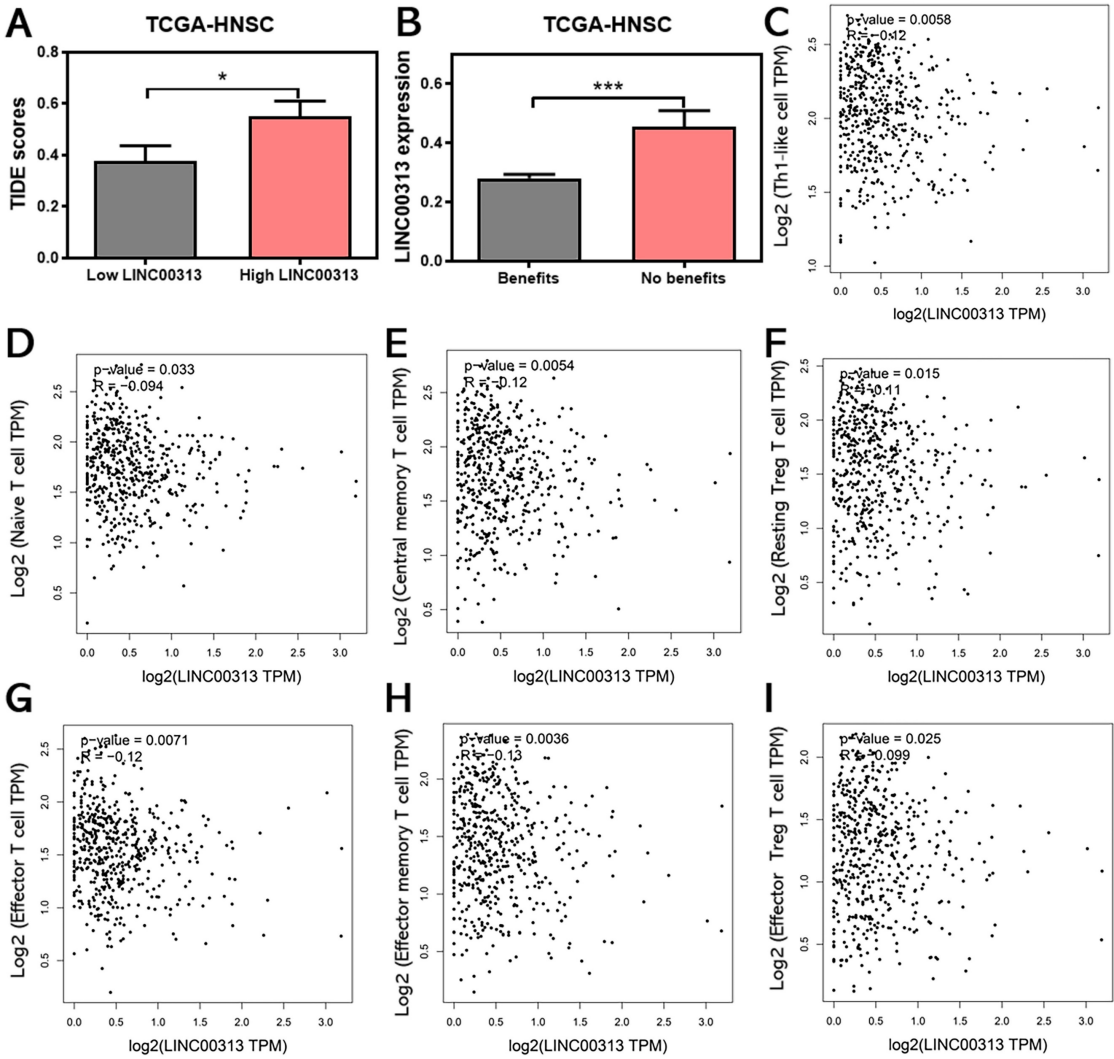
***LINC00313* expression may be associated with HNSC immunotherapy response. (A)** Correlation between *LINC00313* and the TIDE score. **(B)** The correlation between *LINC00313* and immunotherapeutic response in HNSC patients. **(C)** *LINC00313* expression in HNSC samples was negative with expression of Th1-like cell, **(D)** Naïve T cell, **(E)** Central memory T cell, **(F)** Resting Treg T cell,** (G)** Effector T cell, **(H)** Effector memory T cell, **(I)** Effector Treg T cell. All analyses based on the TCGA-HNSC dataset. *: P-value < 0.05, ***: P-value < 0.001.

The infiltration of immune cells in tumors often correlates with the outcome of anti-cancer immunotherapy. Therefore, we also predicted the correlation between *LINC00313* expression and the immunotherapeutic response in HNSC patients. The TIDE score was used to predict potential immunotherapy responses, which can well evaluate the efficacy of immunotherapy, such as anti-CTLA4 therapy. The results of the analysis showed that the higher TIDE scores were also found in the *LINC00313* high-expression group based on TCGA-HNSC (P<0.05)** (Figure 7A).**

Subsequent analysis showed that the expression of *LINC00313* was significantly higher in patients who could not benefit from immunotherapy than in those who could (P<0.001) **(Figure 7B)**. The analysis based on the ICGC-HNSC dataset showed the same trend** (Supplementary Figure 5).** Currently, the two top pillars of immunotherapy in oncology treatment: PD-1/PD-L1 inhibitors and CAR-T therapies are both born out of T-cell-based approaches. T cells play an irreplaceable role in the tumor immune microenvironment, which concerns whether immunotherapy can benefit patients, so we analyzed the correlation between *LINC00313* expression and markers of different T cell subtypes (Th1-like cell: *CXCL13, HAVCR2, IFNG, CXCR3, BHLHE40,* and* CD4*; Naive T cell: *CCR7, LEF1, TCF7,* and *SELL*; Central memory T cell: *CCR7, SELL,* and *IL7R*; Resting Treg T cell: *FOXP3*, *IL2RA*; Effector T cell: C*X3CR1, FGFBP2,* and *FCGR3A*; Effector memory T cell: *PDCD1, DUSP4, GZMK, GZMA*, and *IFNG*; Effector Treg T cell: *FOXP3, CTLA4, CCR8,* and *TNFRSF9*).

The results showed that the expression of *LINC00313* was negatively correlated with the expression of all the above-mentioned T cell subtypes **(Figure 7C-I)**. This is a very meaningful finding, suggesting that *LINC00313* may play an important role in the immune microenvironment of HNSC tumors. Taken together, these results suggest that *LINC00313* maybe a predictive target for immunotherapy response.

### *LINC00313* genomic variants are associated with clinical characteristics of HNSC patients

Non-coding RNAs frequently undergo genome-level mutations. We analyzed the *LINC00313* copy number variation based on two cohort data from TCGA database (TCGA, PanCancer Atlas; TCGA, Firehose Legacy) and found gene altered in 0.57% **(Supplementary Figure 6A)**. We also analyzed the correlation between the *LINC00313* copy number variations and neo-tumorigenesis after the first treatment in HNSC patients, and found that a higher proportion of patients with altered *LINC00313* copy number experienced neoplastic events compared to those without copy number alterations** (Supplementary Figure 6B).** Furthermore, the altered *LINC00313* copy numbers were significantly associated with lower disease-free survival rates (P=0.0445)** (Supplementary Figure 7).** Waterfall plot showing genomics alteration correlation with *LINC00313* expression **(Supplementary Figure 8).** We found a greater probability of gain mutations at loci *7p11.2*,* 8q11.21*, *8q24.21*, *11p13*, *11q13.3*, and* 13q34* in the *LINC00313* high-expression group.

**Figure 8:**
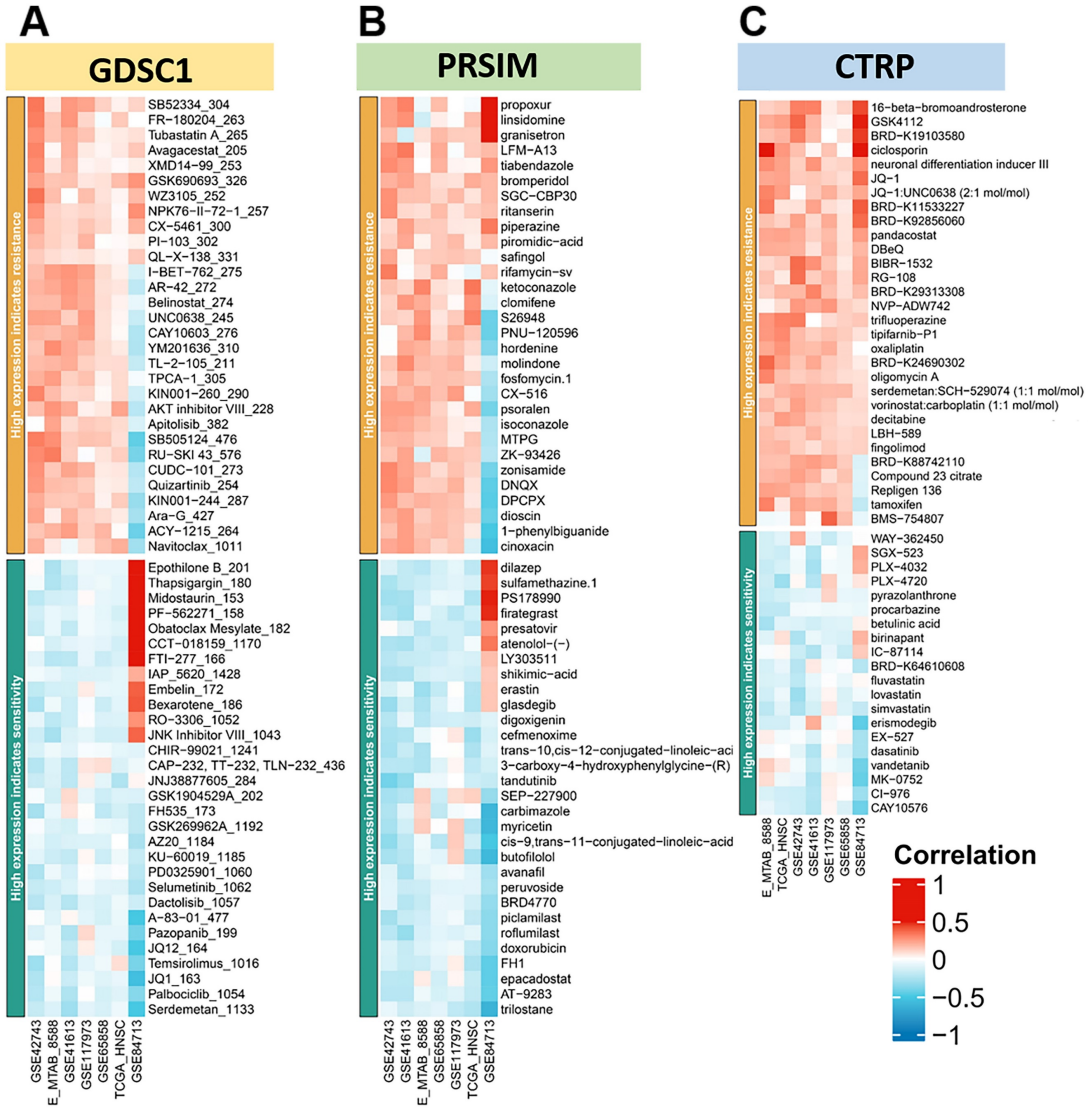
***LINC00313* expression for prediction of drug efficacy*.* (A-C)** The heat map of high expression *LINC00313* showing sensitivity and resistance to chemotherapeutic drugs, based on the GDSC1, PRSIM, and CTRP databases, respectively.

In addition, there was a higher probability of loss mutations at locus *7q31.1*, *8p23.2*, *18q23*, and *19p13.3*. Finally, we plotted the heat map of *LINC00313* high-expression showing sensitivity and resistance to chemotherapeutic drugs according to GDSC1 (Genomics of Drug Sensitivity in Cancer), PRSIM (Profiling relative inhibition simultaneously in mixtures), and CTRP (The Cancer Therapeutics Response Portal) databases **(Figure 8A-C)**. We found that the *LINC00313* high-expression group showed resistance to many chemotherapeutic drugs. Meanwhile, antitumor drug resistance had positive correlations with the expression of *LINC00313*. Based on analysis of common drug databases, no sensitive drugs were shown to target the *LINC00313* high-expression group.

## Discussion and Conclusion

*LINC00313* is upregulated in various cancers including osteosarcoma, lung cancer, thyroid cancer, cervical cancer, etc. Likewise, we found that *LINC00313* expression was significantly higher in the HNSC compared to normal tissues. In addition, patients with high *LINC00313* expression levels had lower overall survival and recurrence-free survival, suggesting that high *LINC00313* expression is associated with poor prognosis in HNSC. Based on several databases analyses of anti-tumor drugs, we found that high *LINC00313* expression was positively correlated with the resistance of many anti-tumor drugs. This could also indirectly indicate a correlation between high *LINC00313* expression and poorer prognosis. In addition, we found for the first time that genomic variants in *LINC00313* were significantly associated with the clinicopathological characteristics of HNSC. The patients with altered *LINC00313* copy numbers had a higher incidence of post-treatment neoplastic events and lower disease-free survival. Thus, genomic variants of non-coding genes may also play an essential role in HNSC and should be explored further as potential biomarkers.

Several recent studies have shown that EMT is critical to the progression of HNSC 38, and is regulated by lncRNAs 39, 40. The pro-EMT effects of *LINC00313* have also been reported for cervical squamous cell carcinoma 41 and thyroid cancer 42. In our study, we found that *LINC00313* silencing inhibited the migration of HNSC cells and *LINC00313* expression correlated significantly with that of the EMT signaling pathways. In addition, a positive correlation was observed between *LINC00313* and mesenchymal markers as well as EMT-promoting transcription factors, whereas the epithelial markers were negatively correlated with *LINC00313*. These data suggest that *LINC00313* may be associated with EMT signaling activation in HNSC cells, and silencing *LINC00313* may inhibit HNSC metastasis. Our study further underscores *LINC00313* potential as a therapeutic target in various tumors. However, the molecular mechanisms underlying its action require further investigation.

Surgery, conventional radiotherapy, and chemotherapy have been the mainstay of HNSC treatment. In recent years, immune checkpoint-based immunotherapy has gradually emerged as an alternative treatment modality for HNSC 43. Immune checkpoint molecules such as PD-L1^44^ and the degree of infiltration of immune cells are predictors of immunotherapy response in cancer patients 45. By exploring the downstream signals regulated by *LINC00313*, we also found that *LINC00313* is well correlated with immune-related signaling pathways, such as “Primary Immunodeficiency”, “IL-17 signaling pathway”, and “B cell receptor signaling pathway”. Our result showed that *LINC00313* correlated negatively with the infiltration of B cells, CD8+ T cells in HNSC tumors. The expression level of *LINC00313* was significantly negatively correlated with immune checkpoints BTLA, CD8A, and CD19. Most importantly, we analyzed the correlation of *LINC00313* expression with markers of different T-cell subtypes, and the results showed that *LINC00313* expression was negatively correlated with almost all T-cell subtypes. These suggests that high *LINC00313* expression may be associated with a "cold" tumor, which has no or few immune cells in tumor tissue 46. These results suggest that tumor immune escape may be involved in *LINC00313*-mediated HNSC development. Interestingly, *LINC00313* expression was significantly lower in patients who could benefit from immunotherapy, and correlated positively with TIDE scores. Taken together, *LINC00313* is a potential marker of immune cell infiltration and immunotherapy response in HNSC.

In this study, we employed online and public databases to analyze the relationship between *LINC00313* expression and the immune response to HNSC, as well as its association with the clinical characteristics of HNSC patients. However, it is important to acknowledge that our conclusions are based solely on bioinformatic analyses and lack experimental validation. Therefore, the exact mechanisms by which *LINC00313* exerts its effects remain to be elucidated through further experimental studies. Additionally, while our findings suggest a potential role for *LINC00313* in the function of HNSC, *in vivo* animal experiments are necessary to confirm these effects. Finally, the clinical therapeutic impact and prognostic value of *LINC00313* in HNSC require further validation through multi-center, large-scale clinical trials. These future investigations will contribute to a more comprehensive understanding of the role of *LINC00313* in HNSC and its potential as a biomarker or therapeutic target. The findings from our study underscore a critical role for LINC00313 in the progression of HNSC, with its upregulation correlating with adverse patient outcomes. We have demonstrated that the downregulation of LINC00313 impedes the migratory capacity of HNSC cells, suggesting its involvement in metastatic processes. Moreover, our data indicate that LINC00313 modulates the tumor immune microenvironment by suppressing immune cell infiltration and immune checkpoint molecule expression, thereby potentially contributing to immune evasion. These insights position LINC00313 as a promising candidate for therapeutic intervention and a biomarker for prognostication in HNSC management. Nevertheless, it is imperative to conduct further basic research and translate these findings into clinical settings to substantiate the therapeutic potential and validate its utility as a prognostic indicator in HNSC patients 47.

## Supplementary Material

Supplementary figures.
