# Supplementary material for: Biomarker Potential of LINC00313 in Head and Neck Squamous Cell Carcinoma: Correlation with Epithelial-Mesenchymal Transition and Immune Cell Infiltration
Source: Int J Med Sci. 2024 Mar 31;21(5):921–36. doi: 10.7150/ijms.93044 (PMC11008489; doi:10.7150/ijms.93044)
Supplement: Supplementary file 1 — Supplementary figures. [file ijmsv21p0921s1.pdf]

Supplementary Material

Biomarker Potential of *LINC00313* in Head and Neck Squamous Cell Carcinoma: Correlation with Epithelial-Mesenchymal Transition and Immune Cell Infiltration

Yikun Ju[1]†, Fang Zhu[2]†, Bairong Fang[1*]

†These authors share first authorship.

*Corresponding Author: Bairong Fang, Department of Plastic and Aesthetic (Burn) Surgery, The Second Xiangya Hospital, Central South University, Changsha, 410011, China. Email: fbrfbr2004@csu.edu.cn

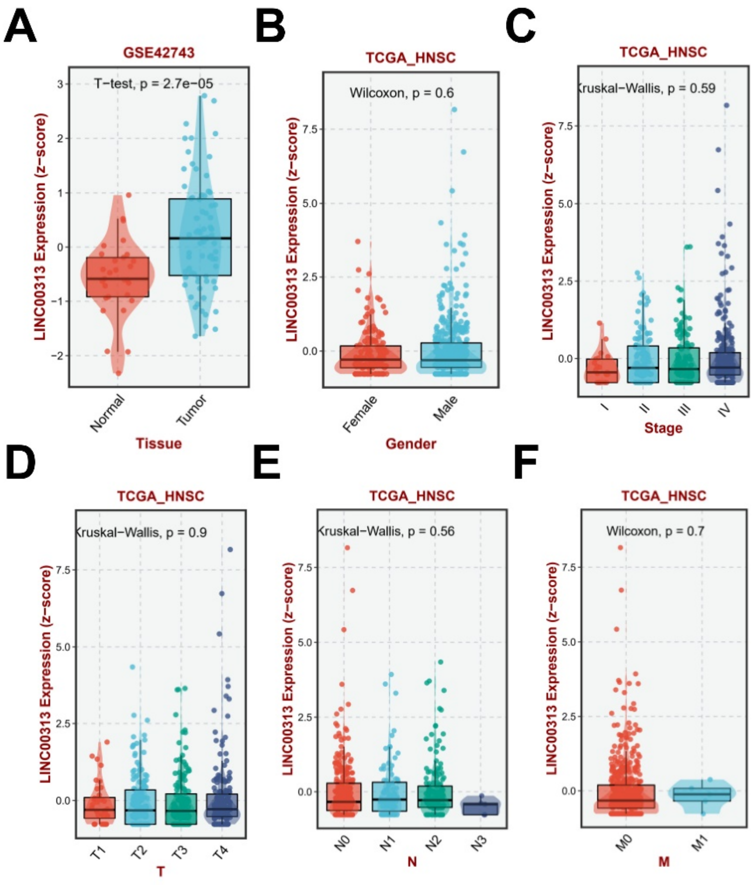

Figure S1. Association between LINC00313 and clinical features. **(A)** LINC00313 expression in HNSC and normal tissues based on the GEO GSE42743 dataset. **(B)** Expression of LINC00313 in HNSC patients of different genders. **(C)** Expression of LINC00313 in HNSC patients of different stages. **(D)** Expression of LINC00313 in HNSC patients of different T stages. **(E)** Expression of

LINC00313 in HNSC patients of different N stages. **(F)** Expression of LINC00313 in HNSC patients of different M stages.

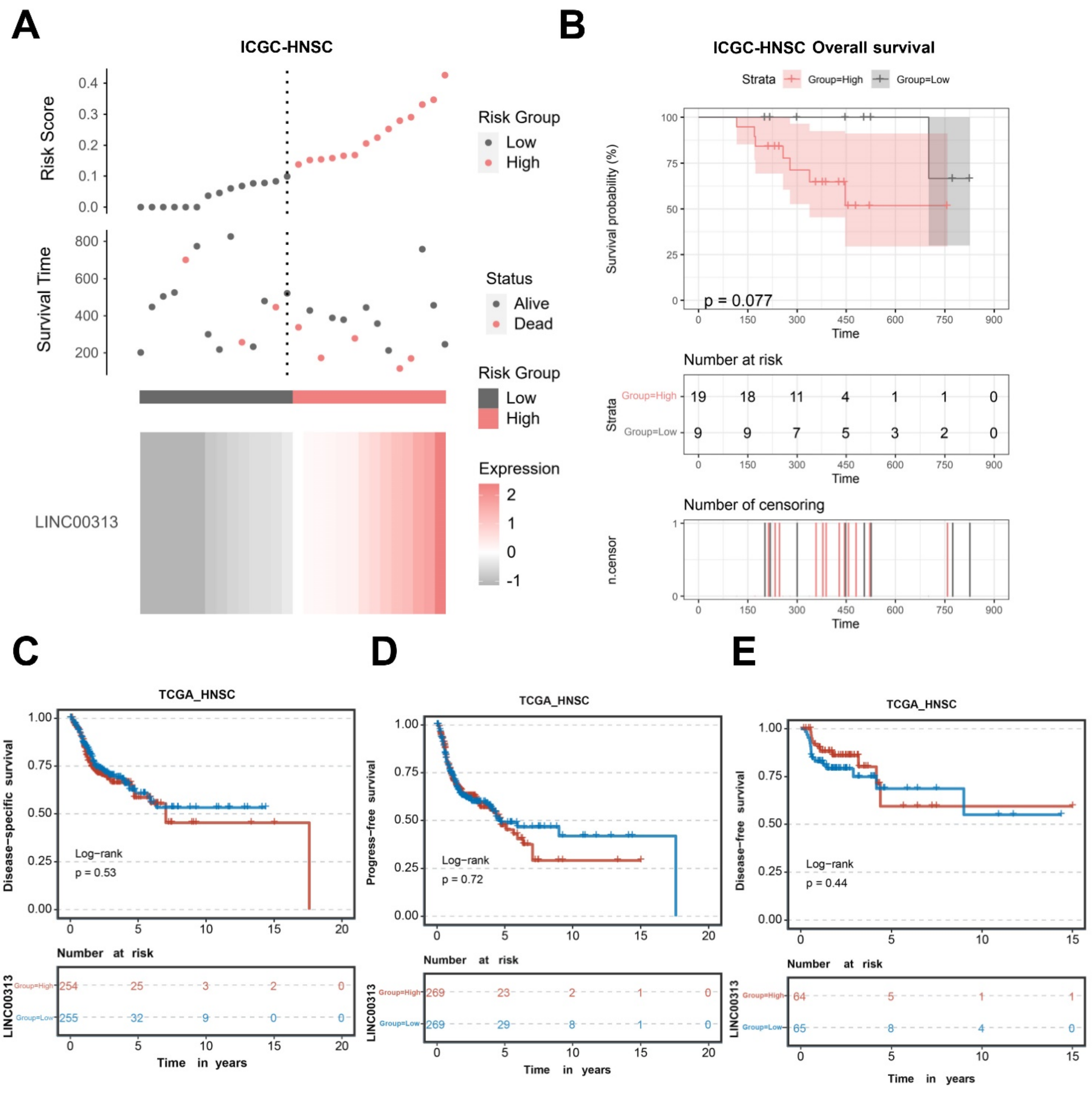

Figure S2. (A) Risk score curve showing survival of patients and expression profiles of LINC00313 in low- and high-risk groups based on ICGC-NHSC dataset. **(B)** LINC00313 expression correlated with the overall survival rates based on ICGC-NHSC dataset. **(C, D, E)** The association between LINC00313 and DSS, PFS, DFS of NHSC patients based on TCGA-HNSC dataset.

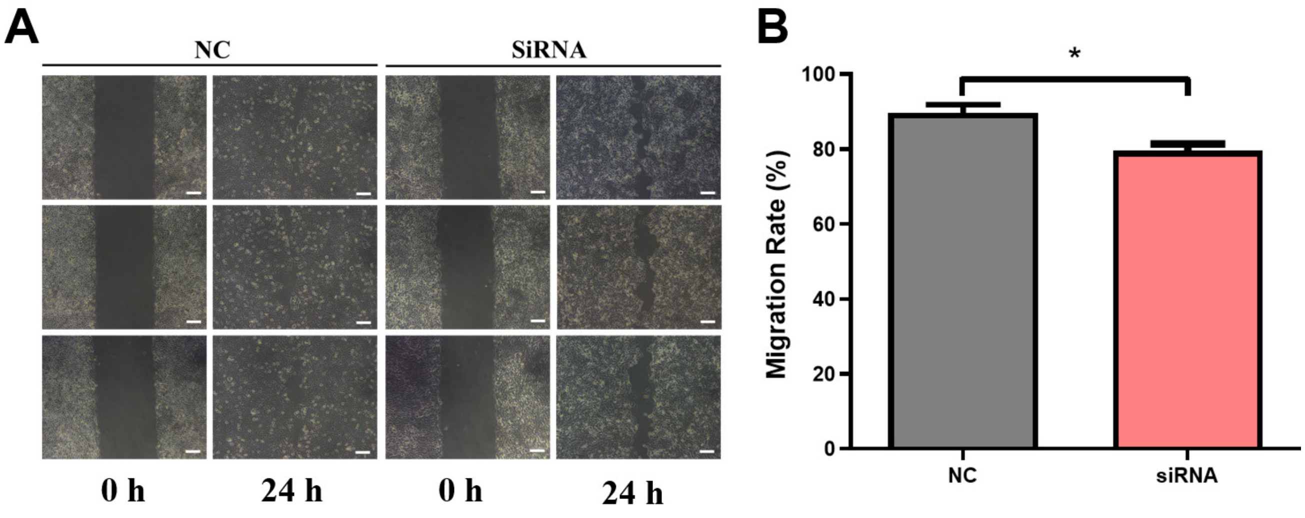

Figure S3. (A&B) Cell scratch migration experiment. The scale bar is 100μm. *:P-value < 0.05

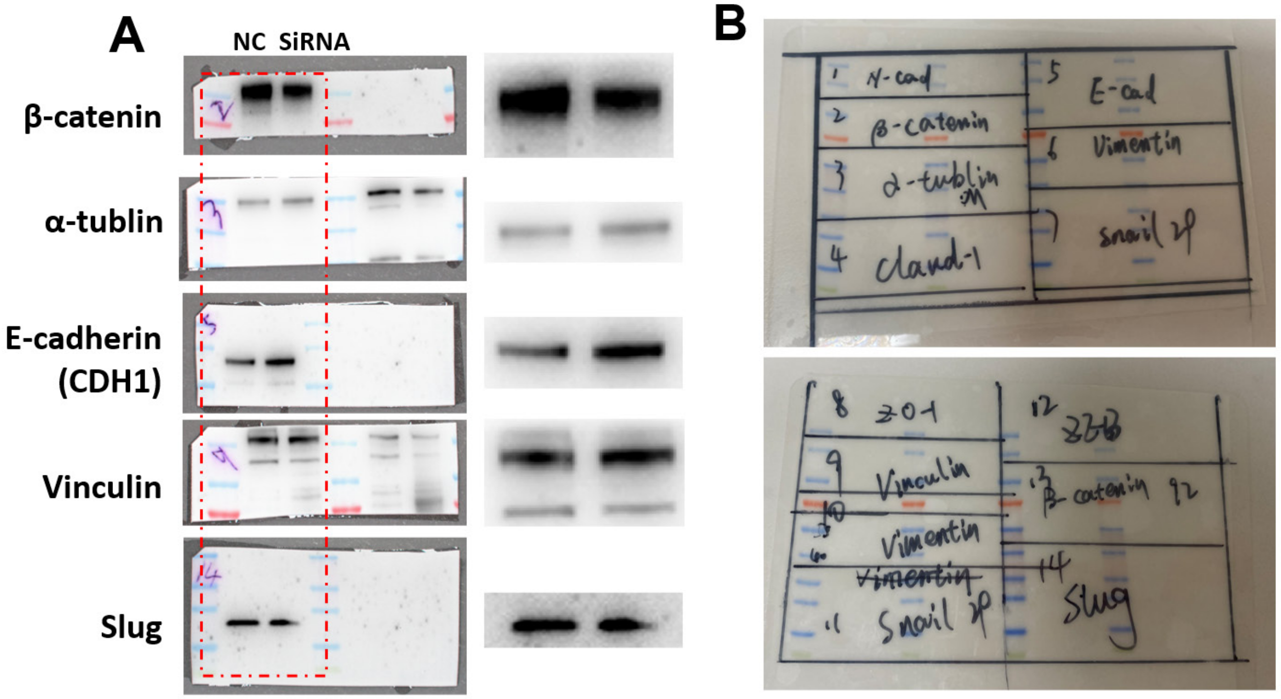

Figure S4. Western Blot raw data. (A) The same batch of transfected SCC-9 cells, after obtaining the precipitate, were subjected to WB experiments. The experimental results were obtained from two gels and two PVDF membranes, with the same amount of supersampling in each column. **(B)** Photo and diagram of PVDF membranes before cutting.

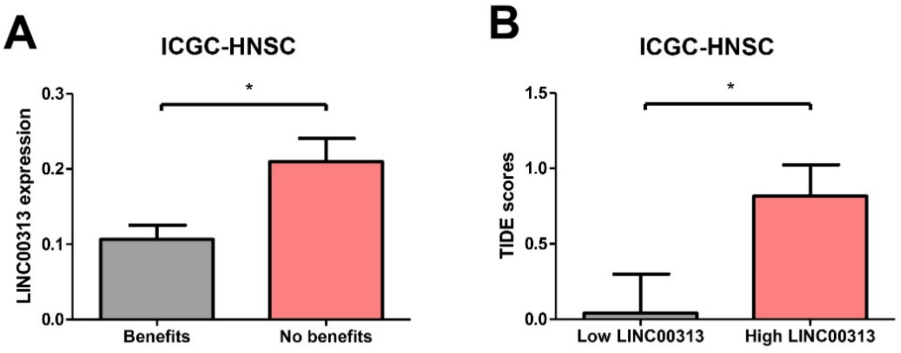

Figure S5. (A) Correlation between LINC00313 and the TIDE score based on ICGC-HNSC dataset. **(B)** The correlation between LINC00313 and immunotherapeutic response in HNSC patients based on ICGC-HNSC dataset. *:P-value < 0.05

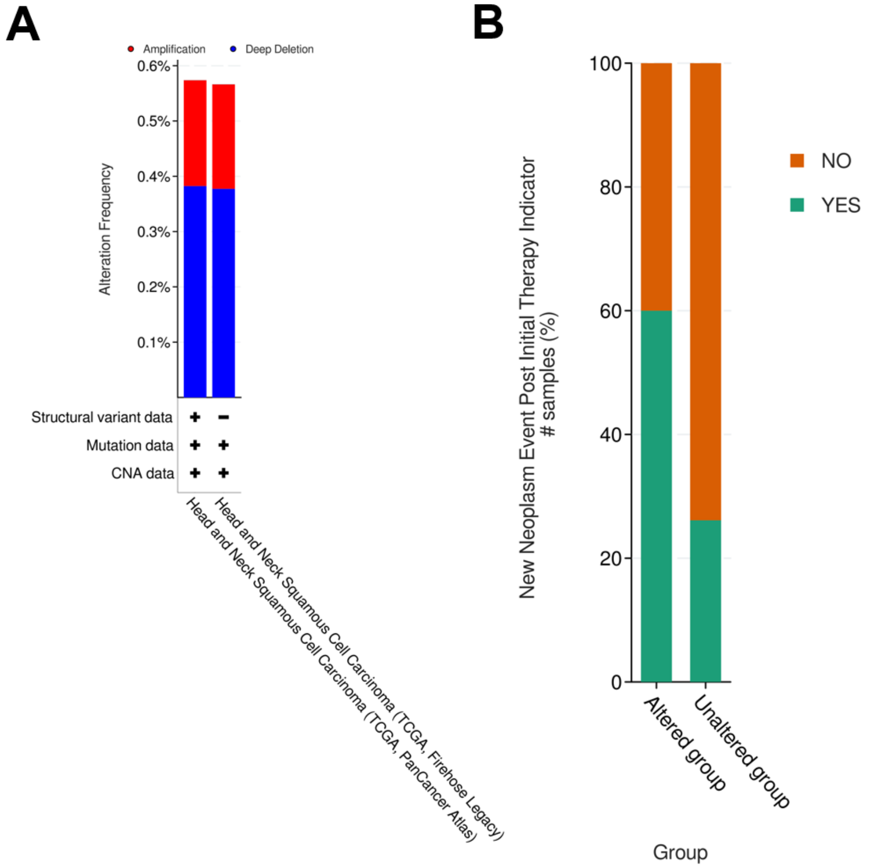

Figure S6. (A) The copy number of LINC00313 was differentially amplified or deleted in the TCGA-HNSC Firehose Legacy dataset and TCGA-HNSC PanCancer Atlas dataset. **(B)** The correlation between the LINC00313 copy number variant and neo-tumorigenesis event after the first treatment in patients with HNSC.

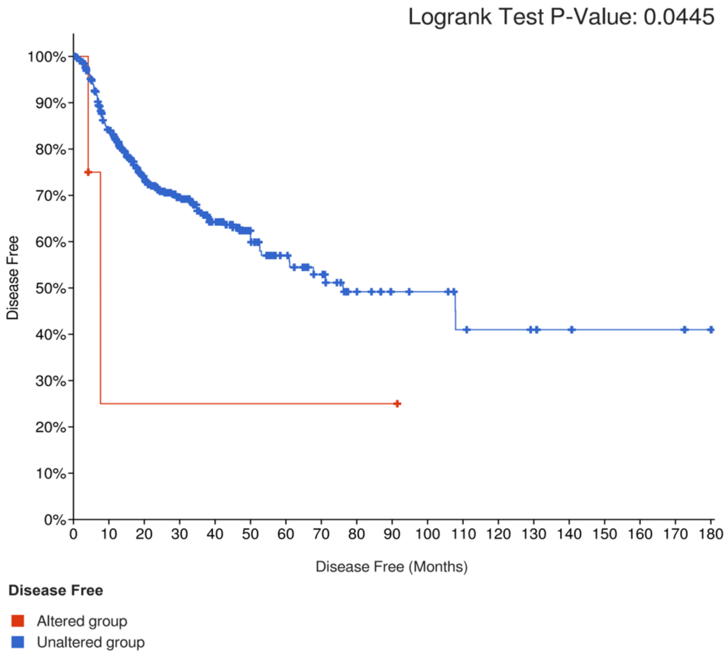

Figure S7. The disease-free rates of patients with altered and unaltered LINC00313 copy number.

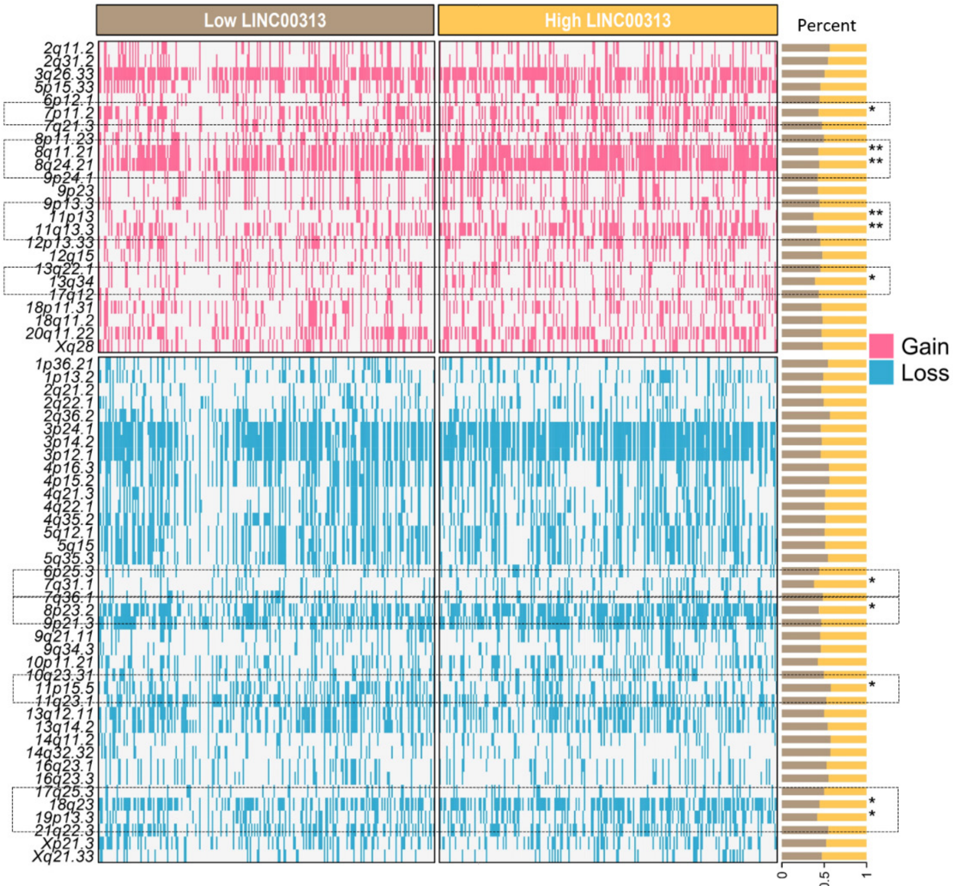

Figure S8. Genomics alteration correlation with LINC00313 expression. *: P-value < 0.05, **: P-value < 0.01.
